# Transcriptome Analysis of CCR9+ T Helper Cells From Primary Sjögren’s Syndrome Patients Identifies CCL5 as a Novel Effector Molecule

**DOI:** 10.3389/fimmu.2021.702733

**Published:** 2021-07-27

**Authors:** Anneline C. Hinrichs, Sofie L. M. Blokland, Ana P. Lopes, Catharina G. K. Wichers, Aike A. Kruize, Aridaman Pandit, Timothy R. D. J. Radstake, Joel A. G. van Roon

**Affiliations:** ^1^Department of Rheumatology & Clinical Immunology, University Medical Center Utrecht, Utrecht University, Utrecht, Netherlands; ^2^Center for Translational Immunology, University Medical Center Utrecht, Utrecht University, Utrecht, Netherlands

**Keywords:** Primary Sjögren’s syndrome, CCR9+ Th cells, transcriptomics, autoimmunity, CCL5

## Abstract

**Introduction:**

CCR9+ Tfh-like pathogenic T helper (Th) cells are elevated in patients with primary Sjögren’s syndrome (pSS) and indicated to play a role in pSS immunopathology. Here we delineate the CCR9+ Th cell-specific transcriptome to study the molecular dysregulation of these cells in pSS patients.

**Methods:**

CCR9+, CXCR5+ and CCR9-CXCR5- Th cells from blood of 7 healthy controls (HC) and 7 pSS patients were FACS sorted and RNA sequencing was performed. Computational analysis was used to identify differentially expressed genes (DEGs), coherent gene expression networks and differentially regulated pathways. Target genes were replicated in additional cohorts.

**Results:**

5131 genes were differentially expressed between CCR9+ and CXCR5+ Th cells; 6493 and 4783 between CCR9+ and CCR9-CXCR5- and between CXCR5+ and CCR9-CXCR5-, respectively. In the CCR9+ Th cell subset 2777 DEGs were identified between HC and pSS patients, 1416 and 1077 in the CXCR5+ and CCR9-CXCR5- subsets, respectively. One gene network was selected based on eigengene expression differences between the Th cell subsets and pathways enriched for genes involved in migration and adhesion, cytokine and chemokine production. Selected DEGs of interest (HOPX, SOX4, ITGAE, ITGA1, NCR3, ABCB1, C3AR1, NT5E, CCR5 and CCL5) from this module were validated and found upregulated in blood CCR9+ Th cells, but were similarly expressed in HC and pSS patients. Increased frequencies of CCR9+ Th cells were shown to express higher levels of CCL5 than CXCR5+ and CCR9-CXCR5- Th cells, with the highest expression confined to effector CCR9+ Th cells. Antigenic triggering and stimulation with IL-7 of the Th cell subsets co-cultured with monocytes strongly induced CCL5 secretion in CCR9+ Th cell cocultures. Additionally, effector CCR9+ Th cells rapidly released CCL5 and secreted the highest CCL5 levels upon stimulation.

**Conclusion:**

Transcriptomic analysis of circulating CCR9+ Th cells reveals CCR9-specific pathways involved in effector T cell function equally expressed in pSS patients and HC. Given the increased numbers of CCR9+ Th cells in the blood and inflamed glands of pSS patients and presence of inflammatory stimuli to activate these cells this suggests that CCR9-specific functions, such as cell recruitment upon CCL5 secretion, could significantly contribute to immunopathology in pSS.

## Introduction

Primary Sjögren’s syndrome (pSS) is a systemic autoimmune disorder characterized by lymphocytic infiltration of exocrine glands in association with dryness of eyes and mouth. The lymphocytic infiltrates mainly consist of CD4 T cells, CD8 T cells and B cells and activation of these cells has been linked to immunopathology in pSS (1–4). A hallmark feature of pSS is B cell hyperactivity, reflected by autoantibody production, elevated serum IgG levels and increased risk of lymphoma development (in 5-10% of patients) (1–3). Both the presence of germinal center-like structures (GCs) and a high number of lymphocytic infiltrates in the salivary glands are associated with lymphoma development (5).

T follicular helper (Tfh) cells are characterized by the expression of C-X-C Motif chemokine receptor 5 (CXCR5) and are potent B cell stimulating cells that can reside in GCs in lymph nodes (6). Tfh cells are elevated in salivary glands and peripheral blood of pSS patients correlating with autoantibody levels, disease severity and aberrant memory B cell and plasma cell subsets (7–9). Recently, a novel “Tfh-like” cell subset expressing C-C chemokine receptor 9 (CCR9) instead of CXCR5 was described with similar characteristics as Tfh cells including interleukin (IL)-21 production and ICOS and Bcl-6 expression (10). CCR9+ Th cells are present in secondary lymphoid organs of both mice and humans (11, 12). In humans, these CCR9+ Th cells produce high levels of IFN-γ, IL-17, IL-10 and IL-4 and strongly induce B-cell responses (11, 13, 14). CCR9+Th cells specifically migrate to mucosal sites in response to their ligand, the C-C Motif Chemokine Ligand 25 (CCL25) and are important for mucosal immune homeostasis (15, 16). However, these cells are also indicated to have a function in mucosal inflammation, contributing to inflammatory bowel disease (IBD) (17, 18). Increased numbers of CCR9-expressing cells have been found in the peripheral blood and inflamed intestinal tissue of Crohn’s disease patients as well as elevated CCL25 production at the inflammatory site (19, 20). Inhibition of CCR9+ Th cells decreases intestinal inflammation in an ileitis mouse model. In Crohn’s disease patients, inconsistent results were demonstrated, possibly due to poor pharmacokinetic properties of the small molecule inhibitors used for therapy (21–23).

CCR9+ T cells were also shown to mediate immunopathology in mucosal tissues in accessory organs of the digestive tract in non-obese diabetic (NOD) mice, including the pancreas and salivary glands. The NOD mice spontaneously developed sialadenitis and had infiltration of IL-21-expressing CCR9+ Th cells in the salivary glands (10). In mice, CCL25 gene expression is upregulated in the oral mucosa upon antigenic triggering and during wound healing (24, 25). In addition to this pivotal role for CCR9+ Th cells in experimental Sjögren-like disease, CCR9+ Th cells are enriched in the circulation of pSS patients and both CCR9+ cells and their ligand CCL25 are elevated in their salivary glands (10, 26, 27). CCL25 mRNA is not detectable in healthy human salivary gland tissue, but is upregulated during oral inflammation (28). Interestingly, the CCR9+ Th cell subset shares functional characteristics with the newly described pathogenic T ‘peripheral helper’ (Tph) cells (ICOS+PD-1^hi^CXCR5-) which drive B cell activation in rheumatoid arthritis patients (29). In pSS patients these ICOS+PD-1^hi^CXCR5- Tph cells were identified in elevated numbers both in salivary glands with germinal centers and in peripheral blood, frequently co-expressing IL-21 and IFN-γ (30). CXCR5- Tph cells were found to have low *CCR9* RNA expression (29). Hence, circulating CCR9+ Th cells from pSS patients that express ICOS and PD-1, as was recently described (26), seem to represent a separate population. In addition, CCR9+ Th cells are IL-7Rhi and robustly respond to IL-7 *in vitro* (26). Since the IL-7/IL-7R axis plays an important role in pSS immunopathology and in GC formation this supports a role for CCR9+ Th cells in pSS (31–38).

To investigate which processes may drive CCR9+ Th cell pathogenicity, transcriptomic profiling of circulating CCR9+ Th cells was performed in healthy controls as compared to pSS patients. Here we demonstrate the transcriptome of CCR9-expressing pathogenic T helper cells from primary Sjögren’s syndrome patients and healthy controls and identify CCL5 as a novel effector molecule of CCR9+ Th cells.

## Methods

### Patients and Healthy Controls

For cell sorting and subsequent RNA sequencing, 7 healthy controls (HC) and 7 pSS patients were included. For replication experiments separate cohorts were used. qPCR validation was performed in n=18 HC and n=9 pSS patients and for flow cytometry n=23 HC and n=22 pSS patients were included, respectively. For validation of CCL5 n=24 HC and n=16 pSS patients were included. All pSS patients were diagnosed by a rheumatologist and met the 2002 American-European Consensus Group (AECG) criteria and the ACR-EULAR criteria (39, 40). All healthy volunteers and pSS patients were included in the University Medical Center (UMC) Utrecht. The UMC Utrecht Medical Research Ethics Committee (METC) approved the study (reference 13/697) and all participants gave written informed consent. Demographic and clinical data are shown in **Table 1**.

**Table 1:** Patients’ characteristics.

	RNA sequencing		qPCR		Flow cytometry		CCL5 validation	
	HC (n = 7)	pSS (n = 7)	HC (n = 18)	pSS (n = 9)	HC (n = 23)	pSS (n = 22)	HC (n = 24)	pSS (n = 16)
**Female gender, n (%)**	7 (100)	7 (100)	16 (89)	9 (100)	18 (78)	20 (91)	24 (100)	15 (94)
**Age, years**	44 ± 14	48 ± 12	52 ± 9	56 ± 8	48 ± 11	58 ± 13	51 ± 11	57 ± 10
**Anti–Ro/SSA positive, n (%)**	–	4 (57)	–	8 (89)	–	17 (77)	–	11 (69)
**Anti–La/SSB positive, n (%)**	–	3 (43)	–	6 (67)	–	10 (45)	–	7 (44)
**ANA positive, n (%)**	–	5 (71)	–	8 (89)	–	17 (77)	–	10 (63)
**Lymphocytic focus score (foci/4mm^2^)**	–	3.2 ± 2.5	–	1.2 ± 1.1	–	2.7 ± 1.7	–	2.5 ± 2
**IgA positive plasma cells (%)**	–	43 ± 29	–	59 ± 26	–	47 ± 25	–	57 ± 24
**Schirmer (mm/5min)**	–	11 ± 11	–	6 ± 8	–	4 ± 4	–	1 ± 2
**Serum IgG (g/L)**	–	11.8 ± 2.9	–	18.1 ± 4.5	–	16.5 ± 8.2	–	13.6 ± 4.1
**ESSDAI score (0–123)**	–	7.4 ± 5.7	–	5.0 ± 3.3	–	6.1 ± 4.9	–	4.6 ± 3.2
**ESSPRI score (0–10)**	–	6.4 ± 1.8	–	6.5 ± 1.1	–	6.0 ± 1.8	–	7.0 ± 1.3
**Immunosuppressants, n**	–	5	–	1	–	5	–	5
**Hydroxychloroquine, n**	–	4	–	1	–	2	–	4
**Other, n**	–	1	–	0	–	3	–	4

Mean ± SD are shown unless otherwise specified. pSS, primary Sjögren’s syndrome; HC, healthy controls; ESSDAI, EULAR Sjögren’s syndrome disease activity index; ESSPRI, EULAR Sjögren’s syndrome patient reported index.

### Fluorescence-Activated Cell Sorting (FACS)

**Figure 1:**
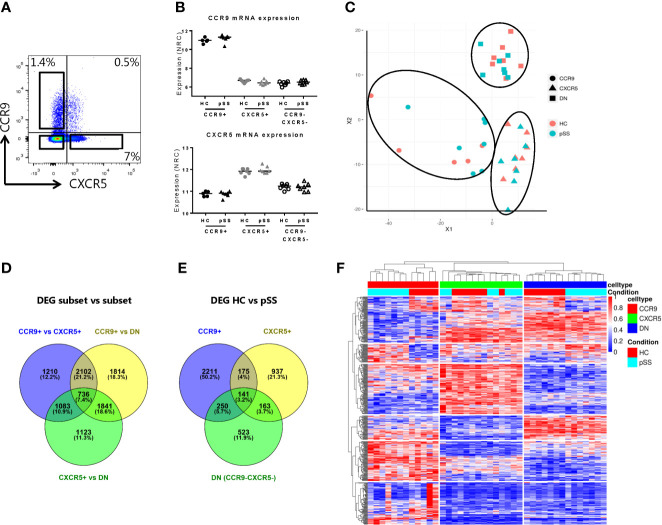
Transcriptomic profiling robustly separates CCR9+, CXCR5+ and CCR9-CXCR5- Th cell subsets and identifies differentially expressed genes in pSS patients. RNA sequencing was performed on FACS sorted CCR9+, CXCR5+ and CCR9-CXCR5- (double negative, DN) T helper cells from PBMCs from healthy donors (HC, n=7, CCR9+ subset n=5) and primary Sjögren’s syndrome patients (pSS, n=7). Representative flow cytometry plot of CCR9 and CXCR5 expression on CD4+ Th cells used for FACS sorting **(A)**. Confirmation of elevated mRNA expression of CCR9 and CXCR5 in the sorted CCR9+ and CXCR5+ Th cells, respectively **(B)**. NRC: normalized read counts, log2 normalized. Multidimensional scaling plot (MDS) shows all the differentially expressed genes (DEGs) between the Th cell subsets **(C)**. Venn diagrams show number and percentages of DEGs between the CCR9+ *vs* CXCR5+ *vs* DN Th cell subsets **(D)** and between HC and pSS patients within the CCR9+, CXCR5+ and DN Th cell subsets **(E)**. Heatmap representation of hierarchical clustering analysis using the top 100 DEGs of all comparisons, shows distinct gene expression profiles between the subsets and between HC and pSS patients **(F)**.

Fresh peripheral blood mononuclear cells (PBMCs) were isolated by Ficoll density gradient centrifugation from Li-heparin blood. PBMCs were stained with fluorochrome-conjugated antibodies against CD3, CD4, CXCR5 and CCR9 (**Supplementary Table 1**). CCR9+CXCR5- (CCR9+), CXCR5+CCR9- (CXCR5+) and CCR9-CXCR5- (double negative, DN) Th cells were sorted with Fluorescence-activated cell sorting (FACS) using BD FACSARIAIII and harvested into tubes with RPMI 1640 containing 10% FCS and 1% penicillin/streptomycin (gating strategy, **Figure 1A**). Cells were lysed in RLTplus buffer (Qiagen) with 1% beta-mercaptoethanol.

### RNA Sequencing and Computational Analyses

RNA was extracted from the sorted Th cell subsets from HC and pSS patients using the AllPrep Universal Kit in QIACube (both Qiagen). Two CCR9+ Th cell samples from healthy controls had to be excluded from analyses since they failed the quality control.

RNA sequencing libraries were generated with the TruSeq RNA Library Prep Kit (Illumina) and sequenced on an Illumina HiSeq4000 generating approximately 20 million 150bp paired-ended reads. The sample qualities were assessed by FastQC. The sequencing reads were aligned to human genome (GRCh38 build 79) using STAR aligner (41). For all the samples >90% of the reads were uniquely mapped to the human genome.

HTSeq-count was used to generate read counts. To obtain normalized read counts (NRC), which are log2 normalized, we performed the variance-stabilizing transformation (VST) on the raw read counts using R/Bioconductor package DESeq2 (42). To identify differentially expressed genes between Th subsets, paired analyses were performed using likelihood ratio test (LRT). Differential expression between HC and pSS was based on Wald test. Venny (http://bioinfogp.cnb.csic.es/tools/venny/) was used to generate Venn diagrams. Genes were considered differentially expressed with a nominal p-value <0.05. Differentially expressed genes (DEGs) from all comparisons were selected for further analyses. No filtering of genes was performed based on read counts, as all samples had similar gene count distributions. Multidimensional scaling (MDS) plot was generated using R function ‘cmdscale’. Hierarchical clustering based on Euclidean distances was performed in R using the top 100 DEGs from all comparisons. Using weighted gene co-expression network analysis (WGCNA) we constructed fifteen gene co-expression networks (modules). WGCNA parameters: soft threshold power = 7, network type = unsigned, correlation method = Spearman, minimum module size = 30, cutHeight or maximum dissimilarity = 0.25. For each module pathway enrichment analysis was performed using ToppFun (https://toppgene.cchmc.org/enrichment.jsp), with FDR corrected p<0.05. The categories GO: molecular function, GO: biological process and pathway were considered.

Target genes for validation were selected from the modules of interest using criteria including expression level (normalized read counts >8), module membership >0.7, size of differential expression between Th subsets (FC >1.5) and between HC and pSS (FC >1.5 or <0.7).

### Gene Expression Replication by qPCR

To validate RNA sequencing results, TaqMan assays were performed for selected target genes (*ITGA1* Hs00235006_m1, *C3AR1* Hs00269693_s1, *ABCB1* Hs00184500_m1, *CCL5* Hs00982282_m1, *SOX4* Hs04987498_s1 and *HOPX* Hs04188695_m1, and *B2M* Hs00187842_m1, all from ThermoFisher, LifeTechnologies) using RNA extracted from FACS sorted CCR9+, CXCR5+ and CCR9-CXCR5- Th cell subsets from HC and pSS patients as described above. RNA was extracted using the AllPrep Universal Kit (Qiagen), according to manufacturer’s instructions. RNA was quantified using NanoDrop and cDNA was constructed using Superscript (Invitrogen). cDNA was measured with the specific TaqMan assay on the Quantstudio 12k flex System using the TaqMan Fast Advanced master mix (LifeTechnologies). Relative mRNA expression was calculated according to the comparative threshold cycle, using *B2M* as endogenous control. The fold change (FC) was calculated using the mean of the CCR9-CXCR5- Th cell subset from the HC as a reference.

### Gene Expression Replication by Flow Cytometry

For phenotypic validation of RNA sequencing results fresh PBMCs were stained with fixable viability dye (eBioscience) and fluorochrome-conjugated antibodies against CD3, CD4, CXCR5, CCR9, NCR3 (NKp30), CD8, CD56, CD73 (NT5E), CD45RO, CD27, CD103 (integrin αE), CD49a (integrin α1), ABCB1 (MDR-1, P-glycoprotein, CD243) and CCR5 (**Supplementary Table 1**). ABCB1 and CCL5 were stained intracellularly using the Fixation-Permeabilization protocol from the manufacturer (eBioscience). CCL5 expression was quantified intracellularly upon culture for 4 hours with or without stimulation with PMA and ionomycin in the presence of Brefeldin A. This protein secretion inhibitor was added as in part of the analyses IFN-γ and TNF-α expression was assessed to identify CCL5 expression by IFN-γ/TNF-α -producing effector Th cells. Importantly, in contrast to IFN-γ and TNF-α, CCL5 is hardly affected by inhibitors of cytokine secretion such as Brefeldin A (43). Thus T cell stimulation results in CCL5 release reflected by reduced intracellular expression and subsequently release was expressed as the difference (delta) of mean fluorescence intensity (MFI) from Th cells with and without T cell stimulation (PMA/ionomycin).

### Culture

For analysis of CCL5 secretion, 2.10^4^ CCR9+, CXCR5+ or CCR9-CXCR5- FACS sorted Th cells were cultured with 5.10^3^ monocytes (MACS sorted with CD14 beads) for 3 days with 10 ng/mL IL-7 or 0.1 ng/mL superantigen (Staphylococcal enterotoxin B, SEB) and restimulated with PMA and ionomycin for 24h. CCL5 release in the supernatants was measured by Luminex technology as previously described (44).

### Statistical Analysis

RNA sequencing data were analyzed as described above. For the validation experiments (flow cytometry, qPCR and CCL5 production) Student’s t-test, paired parametric t-test, Mann-Whitney U test and Wilcoxon non-parametrical paired test were used where appropriate. Data were analyzed using FlowJo™ Software, Graphpad Prism 6 and IBM SPSS Statistics 26. Differences were considered statistically significant at p ≤ 0.05.

## Results

### Transcriptome Analysis Identifies Differentially Expressed Genes Between Th Cell Subsets and Between Healthy Controls and pSS Patients

To validate our technical procedure of sorting CCR9+, CXCR5+ and CCR9-CXCR5- (double negative, DN) Th cells and RNA isolation procedure, we confirmed elevated *CCR9* mRNA expression in the CCR9+ Th subset and elevated *CXCR5* mRNA expression in the CXCR5+ subset (**Figures 1A, B**). The isolated Th subsets were robustly distinguished based on their transcriptomic profile as shown in the multidimensional scaling (MDS) plot in **Figure 1C**. 5131 genes were differentially expressed between CCR9+ and CXCR5+ Th cells; 6493 genes were differentially expressed between CCR9+ and DN and 4783 genes were differentially expressed between CXCR5+ and DN (**Figure 1D**). Transcriptomic profiles differed between HC and pSS, with the largest number of DEGs in the CCR9+ subset, followed by the CXCR5+ subset and the DN subset (2777, 1416 and 1077, respectively **Figure 1E**). Based on the top 100 of DEGs from all comparisons, the Th cell subsets clearly cluster together per subset using hierarchical clustering analysis and most of the samples from patients and controls also cluster separately (**Figure 1F**). These results indicated that gene expression of circulating CCR9+, CXCR5+ and DN Th cell subsets differ in their transcriptomes and that the transcriptomes of HC and pSS patients differ.

### Network Analysis Reveals Th Subset- and Disease-Associated Modules Enriched for Differentially Expressed Pathways

**Figure 2:**
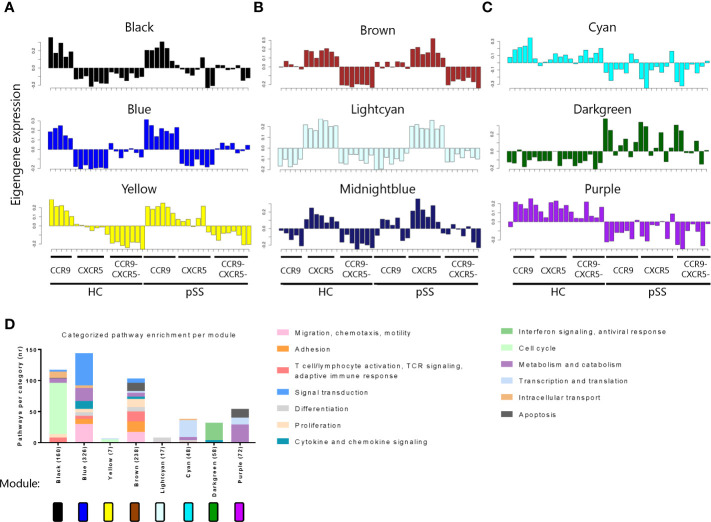
Weighted gene co-expression network analysis (WGCNA) reveals networks of genes with coherent expression patterns involved in distinct Th cell functions. Modules with elevated eigengene expression in CCR9+ Th cells **(A)** or CXCR5+ Th cells **(B)**, and modules with differential eigengene expression between HC and pSS patients **(C)** are shown. Each bar represents the eigengene expression of one sample in the indicated Th cell subset of a donor. Pathway enrichment analysis reveals different pathways within the modules. Pathways are categorized, and categories are indicated by the colors in the legend. The number of pathways per category is shown. The total number of pathways per module is indicated behind the module name on the X-axis **(D)**.

Next we used weighted gene co-expression network analysis (WGCNA) to cluster DEGs into 15 different gene correlation modules each containing a set of genes exhibiting coherent expression patterns. Each module was color named. We further selected modules with the consensus expression pattern (eigengene expression) of interest, for example, modules that revealed gene expression which was strikingly different between the Th subsets or between pSS and HC. As a result, 9 modules were selected: 3 modules with elevated eigengene expression in CCR9+ Th cells (black, blue and yellow, **Figure 2A**) or in CXCR5+ Th cells (brown, lightcyan and midnightblue, **Figure 2B**), and modules with the most differential eigengene expression between HC and pSS (cyan, darkgreen and purple, **Figure 2C**). 6 modules were excluded based on lack of clearly distinct patterns between subsets or between HC and pSS (**Supplementary Figure 1**).

To functionally annotate each module, we performed pathway enrichment analysis. The modules distinguishing between CCR9+ and CXCR5+ Th cells mainly contained pathways involved in cell cycle (black and yellow), but also in effector T cell functions including migration and adhesion, proliferation, T cell activation and TCR signaling and cytokine and chemokine signaling (blue and brown). The modules showing the most distinct patterns between HC and pSS patients, mainly comprised of genes involved in transcription and translation, interferon signaling and metabolism (cyan, darkgreen and purple, respectively) (**Figure 2D**). In the midnightblue module no enrichment for pathways was found, therefore it is excluded from the graph.

### Differentially Expressed Genes Between CCR9+ Versus CXCR5+ and CCR9-CXCR5- Th Cells Reveal Genes Involved in Effector T Cell Function and Transcription Factors Associated With Th1 Differentiation

**Figure 3:**
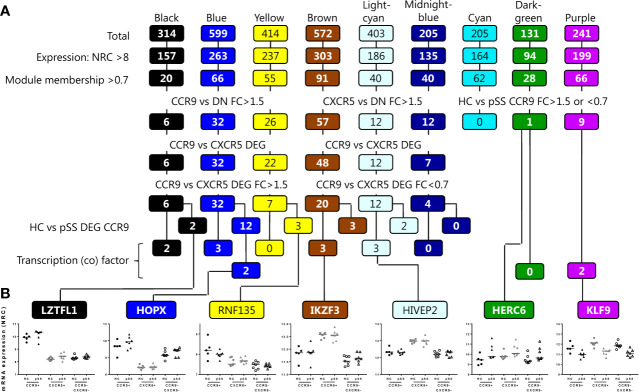
Workflow for selection of target genes for validation per module. Genes from the modules are selected based on the criteria shown, including expression level (normalized read counts >8), module membership >0.7, differential expression between the Th cell subsets with a fold change of >1.5, or between HC and pSS of >1.5 or <0.7, and finally transcription (co) factors are selected. From the lower 4 rows, target genes that are also supported by literature evidence are selected for further analysis **(A)**. Representative examples of genes selected based on the criteria and per module are shown **(B)**.

To narrow down to potential key genes from the RNA sequencing analysis as candidates for validation, a set of genes from the 9 modules was selected. The selection was focused on both the most robust differences between the Th subsets and between HC and pSS. Genes were selected based on several data-driven criteria including expression level (normalized read counts >8), module membership >0.7: indicating a strong correlation of the gene expression profile with the eigengene expression of the module (45), differential expression between the Th cell subsets with a fold change of >1.5 or <0.7, or between HC and pSS of >1.5 or <0.7, finally transcription (co) factors were selected (**Figure 3A**). Representative genes selected from the separate modules following this procedure are shown in **Figure 3B**.

**Figure 4:**
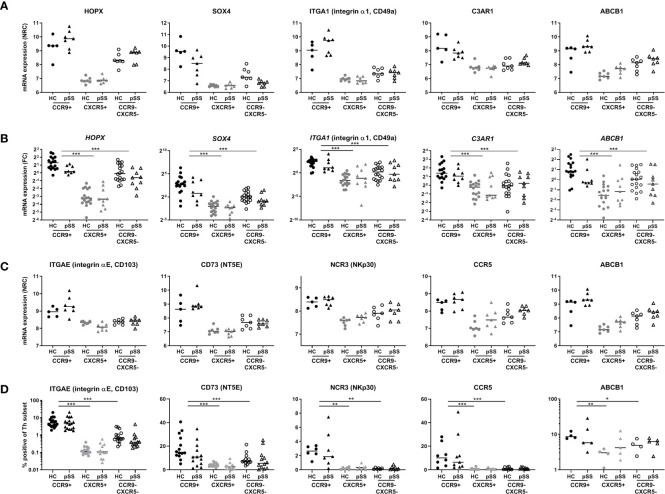
Validation of selected target genes from the blue module in replication cohorts confirms differences between Th subsets, but not between HC and pSS patients. According to the criteria shown in **Figure 3** and based on pathway enrichment analysis (**Figure 2**) and literature, genes were selected from the blue module and were replicated by qPCR and flow cytometry. RNA sequencing data is shown in **(A, C)**, qPCR and flow cytometry data are shown in **(B, D)**, respectively. HOPX, SOX4, ITGA1, C3AR1 and ABCB1 were evaluated by qPCR in an additional replication cohort **(B)**. Genes encoding for surface proteins for which antibodies were available were replicated by flow cytometry: ITGAE (CD103), CD73 (NT5E), NCR3 (NKp30), CCR5 and ABCB1 **(D)**. NRC, normalized read counts; FC, fold change, DN HC mean is set at 1. *, **, *** indicates statistical significance of p < 0.05, 0.01, 0.001, respectively.

Following these selection criteria we subsequently focused on genes from the blue module as this is the module that has the highest number of upregulated genes in CCR9+ Th cells and shows enrichment for the largest number of pathways crucial for effector Th cell function. 8 target genes out of 12 (**Figure 3A**) that were differentially expressed in HC *vs* pSS and have a known function based on literature evidence, were subsequently selected for validation. These included two transcription (co) factors *HOPX* and *SOX4*, and in addition *ITGAE, NT5E, C3AR1, CCL5, ITGA1* and *ABCB1*. Additionally from the 20 genes identified in the blue module that were not differentially expressed between HC *vs* pSS, *CCR5* and *NCR3* were selected given their effector function and potential role in pSS (46–48). Next, we tested whether these selected genes could be replicated in an additional validation cohort using qPCR or flow cytometry. All of the selected DEGs upregulated in the CCR9+ Th subset as compared to the other subsets were validated by qPCR: *ITGA1*, *C3AR1, SOX4, HOPX* and *ABCB1*, (CCR9+ *vs* CXCR5+ and CCR9-CXCR5- all p<0.001, **Figures 4A, B**) or by flow cytometry: *ITGAE* (CD103)*, NT5E* (CD73), *NCR3* (NKp30)*, CCR5* and, *ABCB1* (CCR9+ *vs* CXCR5+ and CCR9-CXCR5- all p<0.05, **Figures 4C, D**). However, the differential expression of these genes between CCR9+ Th cells from HC versus pSS patients was not confirmed in the additional cohort, neither on mRNA nor protein level (**Figure 4**).

As for association with clinical parameters we only found a significant correlation of NT5E with the ESSDAI score (r=0.9274, p=0.0079, based on 7 pSS CCR9+ cell subsets). However, this was not validated in the flow cytometry cohort. Other selected target genes did not have significant correlations with ESSDAI score, LFS or serum IgG nor in RNA sequencing, nor in validation cohorts (data not shown). Also when subdividing in low *vs* moderate-high ESSDAI scores (ESSDAI<5 and ESSDAI≥5) no significant differences were found in any of the cohorts.

### CCR9+ Th Cells, and Mostly Effector CCR9+ Th Cells, From Healthy Controls and pSS Patients Produce High Levels of CCL5 and CCR9+ Th Cells Respond More Potently to IL-7 in the Context of Monocytes

**Figure 5:**
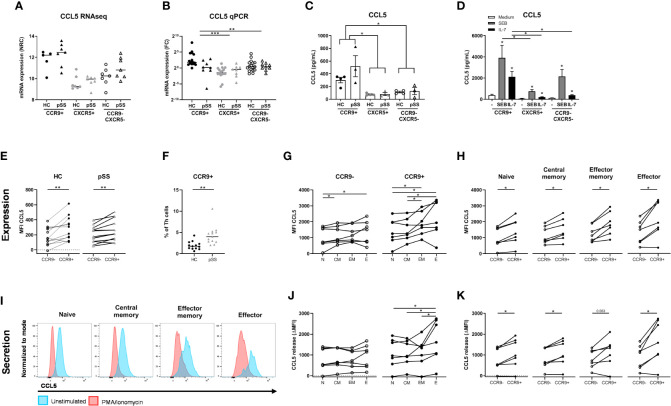
Increased production and release of CCL5 by CCR9+ Th cells in pSS patients and controls is higher in effector cells and is enhanced by IL-7 and antigenic triggering. mRNA expression as assessed by RNA sequencing **(A)**. In a replication cohort qPCR shows validation of significantly increased CCL5 mRNA in the CCR9+ Th cell subset as compared to CXCR5+ and CCR9-CXCR5- Th cell subsets **(B)**. CCR9+, CXCR5+ and CCR9-CXCR5- Th cells from HC (n=4) and pSS patients (n=3) were cultured with monocytes for 72 hours followed by PMA/ionomycin restimulation **(C)**. To determine the effect of antigen triggering (SEB=Staphylococcal Enterotoxin B) and IL-7 stimulation on CCL5 production by the Th cell subsets we pooled HC and pSS **(D)**. CCL5 protein expression in Th cells from peripheral blood from healthy controls (n=13) and pSS patients (n=13) was analyzed ex vivo by flow cytometry in a 2^nd^ replication cohort **(E)**. CCR9 expression on Th cells was also assessed in this replication cohort by flow cytometry **(F)**. In HC CCL5 expression by effector memory and effector Th cells is compared to naive Th cells, both for CCR9- and CCR9+ Th cells **(G)**; and in all CD45RO/CD27 compartments CCL5 expression is compared between CCR9- and CCR9+ Th cells **(H)**. Histograms from a representative donor show CCL5 expression upon 4h of stimulation with PMA/ionomycin in the different Th cell compartments. Blue and red shows CCL5 expression in unstimulated and PMA/Ionomycin stimulated cells, respectively **(I)**. CCL5 release expressed as the difference between unstimulated – the stimulated cultures (delta) between all four CD45RO/CD27 compartments for both CCR9- and CCR9+ Th cells **(J)** and within each compartment comparing CCR9- and CCR9+ Th cells **(K)**. FC: fold change. Th cell phenotypic validation: naive (CD27+CD45RO-), central memory (CD27+CD45RO+), effector memory (CD27-CD45RO+), effector (CD27-CD45RO-) cells. MFI: mean fluorescence intensity. ΔMFI: deltaMFI (unstimulated minus PMA/ionomycin stimulated). *, **, *** indicates statistical significance of p<0.05, 0.01, 0.001, respectively.

*CCL5* (RANTES) is elevated in CCR9+ Th cells at the transcriptomic level compared to the other subsets and met the selection criteria described above (**Figure 5A**). Because of its increased expression in pSS patients and potential key role in regulation of glandular inflammation in Sjogren-like disease in mice (49), CCL5 was studied in more detail using multiple technological platforms. The differential *CCL5* mRNA expression between HC and pSS patients could not be confirmed by qPCR (**Figure 5B**). Nonetheless, differential CCL5 expression between CCR9+ Th cells and the other subsets was replicated (p ≤ 0.01).

Since the number of CCR9+ Th cells and its ligand CCL25 are increased in the blood and salivary glands of pSS patients (10, 26), we analyzed whether CCR9+ Th cells in the context of antigen presenting cells produce CCL5. To test this, FACS sorted CCR9+, CXCR5+ and CCR9-CXCR5- Th subsets were co-cultured with monocytes and CCL5 was measured in supernatants upon PMA/ionomycin restimulation. Significantly elevated levels of CCL5 were measured in the CCR9+ Th cell cultures as compared to CXCR5+ and CCR9-CXCR5- Th cells (p ≤ 0.05, **Figure 5C**). Since antigen triggering and IL-7 stimulation, as previously demonstrated (31, 50) may be key drivers of CCR9+ Th activation and thereby CCL5 secretion, cells were cultured with superantigen Staphylococcal Enterotoxin B (SEB) or IL-7. Interestingly, IL-7 strongly upregulated CCL5 production by co-cultured CCR9+ Th cells. Their CCL5 production is significantly higher than that of co-cultured CXCR5+ or CCR9-CXCR5- Th cells (both p<0.05). SEB even more strongly stimulated CCL5 production by CCR9+ Th cells, significantly higher than CXCR5+ Th cells but not significantly higher than CCR9-CXCR5- Th cells (p=0.18) (**Figure 5D**).

To ensure that CCL5 production is not merely monocyte dependent, using flow cytometry we evaluated CCL5 expression levels directly in CCR9+ Th cells *vs* CCR9- Th cells in blood from HC and pSS patients (gating strategy, **Supplementary Figure 3**). In support of the RNA sequencing data CCR9+ Th cells expressed higher CCL5 levels than CCR9- Th cells, both in HC and pSS patients (p=0.006 and p=0.003, respectively) (**Figure 5E**). In line with the RNA expression data CCL5 protein expression in CCR9+ Th cells in pSS patients was not significantly different from HC. However, we confirmed our earlier findings (26), showing that the frequency of CCR9+ cells in the Th cell subset is significantly higher in pSS patients compared to HC (1.92% ± 0.97 in HC *vs* 3.81% ± 1.25 in pSS patients, **Figure 5F**).

Since CCR9+ Th cells are enriched for effector memory and effector Th cells (26) we tested whether higher CCL5 expression was related to differentiation of Th cells. For this purpose CD45RO/CD27-defined naive, central memory, effector memory and effector Th cell subsets were analyzed.

We here show that CCL5 expression is significantly higher in more differentiated cells (compared to naive Th cells, **Figure 5G**). Also in these separate subsets the difference between CCR9+ and CCR9- Th cells is statistically significantly different (all p<0.05, **Figure 5H**). To determine if more differentiated Th cells release CCL5 more robustly upon stimulation, we compared CCL5 expression in the Th cell subsets after 4 hours PMA/ionomycin stimulation as compared to unstimulated Th cells. Histograms of a representative donor are shown in **Figure 5I**. Release of CCL5 is measured as reduced intracellular CCL5 expression as previously shown (43). Effector Th cells release more CCL5 after stimulation compared to naive, central memory and effector memory cells (all p<0.05, **Figure 5J**). All CCR9+ subsets secrete more CCL5 after stimulation compared to CCR9- cells (p<0.05, except for effector memory cells p=0.063, **Figure 5K**).

Since it has been demonstrated that CCL5 secretion by CD8 T cells is rapidly induced (43) we also assessed whether this holds true for CCR9+ Th cells. For this purpose we performed a separate experiment testing the kinetics of CCL5 release measuring CCL5 at t=0, t=10 minutes and t=4 hours. Interestingly, we found that reduction of CCL5 expression after 10 minutes of stimulation was similar as compared to 4 hour stimulation (reduction at 10 minutes to 61% ± 20 versus 60% ± 24 reduction at 4 hours, p=0.583, n=12 HC). Hence this indicated a rapid release of CCL5 also by CCR9+ Th cells.

Finally, we also assessed whether increased CCL5 expression was a specific feature of IFN-γ/TNF-α -secreting effector Th cells and found that CCR9+ IFN-γ+/TNF-α+ Th cells express higher CCL5 levels (MFI 589 ± 231 *vs* 425 ± 190 compared to CCR9+ IFN-γ-/TNF-α- cells, p=0.018).

## Discussion

In this study, for the first time transcriptomic analysis of CCR9+ Th cells was performed in both healthy controls and pSS patients, and compared to CXCR5+ Th cells. RNA sequencing analysis revealed multiple networks of differentially expressed genes between the Th subsets that were shared between healthy controls and pSS patients. Identified pathways involved in effector T cell function were upregulated in CCR9+ Th cells including genes associated with adhesion, chemotaxis, proliferation, TCR activation, drug response and complement activation. This was exemplified by high production of CCL5 by CCR9+ Th cells as compared to CCR9- Th cells, which was identified in all Th subsets but in particular in effector cells and induced upon antigen challenge and IL-7 stimulation.

Our strategy for selection of target genes to validate, used strict and robust criteria and was focused on upregulated genes in the CCR9+ Th cell subset in one of the CCR9-specific (blue) modules showing significant differences between the Th subsets. Using this strategy we identified a number of differentially expressed genes that were validated in additional cohorts on RNA and/or protein level. These include transcription (co)factors *HOPX* and *SOX4*, chemokine receptor *CCR5*, chemokine *CCL5*, adhesion molecules *ITGAE* (CD103, integrin αE) and *ITGA1* (CD49a, integrin α1), cytotoxicity receptor *NCR3* (NKp30), multidrug resistance gene *ABCB1* (MDR-1, P-glycoprotein), complement receptor *C3AR1* and inhibitory molecule *NT5E* (CD73). Nonetheless, we realize that genes from the other modules potentially also could play key roles in the function of CCR9+ Th cells. For example, *LZTFL1* that has coherence within the black module encodes for a protein that is upregulated by all-trans retinoic acid and upon TCR signaling. Its overexpression has been shown to enhance NFAT mediated signaling, potentially contributing to the production of cytokines by Th cells (51). Similarly, genes from the yellow module include the genes encoding for CCR9 and integrin α4, which have been indicated to represent key molecules for CCR9+ Th cells. Hence, future studies should replicate these genes and reveal their roles. The same holds true for genes that are unique to CXCR5+ Th cells, in particular those that are significantly different between HC and pSS patients. In addition, the interaction between genes in identified networks should be studied. Here we focused on the highly connected and robustly expressed genes in the blue module and their potential role in the pathogenicity of CCR9+ Th cells and their potential relevance to pSS immunopathology is discussed.

Interestingly, we identified transcription cofactor *HOPX* (homeobox only protein) to be strongly expressed by CCR9+ Th cells. *HOPX* previously was shown to be associated with Th1 activity. In humans, *HOPX* is highly upregulated in effector/memory Th1 cells and in mice *HOPX* is crucial for survival of Th1 cells. *HOPX*-deficient mice do not develop colitis or arthritis in models inducing these inflammatory conditions (52). In addition, we demonstrated increased levels of *SOX4* in CCR9+ Th cells as compared to the other Th cells. *SOX4* inhibits Th2 responses and overexpression of *SOX4* during Th1 differentiation induces more IFN-γ-producing cells (53). Together, this suggests that *HOPX* and *SOX4* may drive the high IFN-γ production by CCR9+ Th cells, which we and others have previously shown (13, 26). However, future research is needed to confirm the role of *HOPX* and *SOX4* in regulating Th1-associated activity in CCR9+ Th cells.

In addition to *HOPX* and *SOX4*, *CCL5* and its receptor *CCR5* are upregulated in CCR9+ Th cells and have been associated with a Th1 phenotype (54, 55). Also, CCR5 has been implicated in dry eye disease. In a mouse model of experimental dry eye disease, desiccating stress potently stimulated the expression of Th1-attracting chemokines and their receptors on the ocular surface of C57BL/6 mice (56). In human studies, CCR5 expression has been shown to increase in the conjunctival epithelium of patients with dry eye syndrome (47, 57). Interestingly, CCL5 and its receptor CCR5 were increased in inflamed glands in Sjögren-like disease and blockade of CCL5 can significantly reduce disease (49). The ligands for CCR5, CCL3 and CCL5 are elevated in pSS salivary glands, potentially facilitating recruitment of CCR5-expressing cells including CCR9+ Th cells (48). In this paper we demonstrate higher expression and secretion of CCL5 in CCR9+ effector cells. Also when looking at IFN-γ/TNF-α –secreting cells we find CCR9+ Th cells express highest CCL5. Besides this we have demonstrated rapid release of CCL5 by CCR9+ Th cells and a strongly increased secretion of CCL5 by co-cultured CCR9+ Th cells as compared to CXCR5+ and DN Th cells. Interestingly, IL-7 which is a key early mediator of salivary gland inflammation (31–34) and is a crucial factor in lymphoid structure organization (38, 58), significantly increased CCL5 production by CCR9+ Th cells to a higher level than CXCR5+ and CCR9-CXCR5- Th cells. As IL-7 has previously been shown to increase responsiveness of auto-reactive T cells (59, 60) this could implicate that IL-7-driven self-reactive T cell responses associated with CCL5 production plays a significant role in early inflammatory responses in pSS, attracting multiple leukocyte subsets to affected sites. Similarly, T cell receptor crosslinking by exogenous antigen as we demonstrate in this paper can strongly boost CCL5 production by CCR9+ Th cells.

Genes encoding for adhesion molecules *ITGAE* (CD103) and *ITGA1* (CD49a) were elevated on RNA level in CCR9+ Th cells as compared to the other Th cells. On protein level this corresponded with around 10% of CCR9+ Th cells expressing CD103 which is elevated as compared to the other subsets. CD103 is known as a marker for intraepithelial lymphocytes dimerizing with β7 to form αEβ7, and its ligand is E-cadherin, both of these molecules have been found to be elevated in pSS salivary glands (61). Also, laminin, a ligand for CD49a, has been found to be upregulated in pSS salivary glands (62). However, whereas we did find a subset of CCR9+ Th cells with increased expression of CD103, we could not find any evidence for increased surface expression of CD49a on the CCR9+ Th cells (data not shown). These data indicate that part of the CCR9+ Th cells are prone to adhere in the salivary gland of pSS patients, potentially mediated *via* ITGAE.

In the present study we demonstrate that *NCR3* gene expression is elevated in CCR9+ Th cells. The ligand for NCR3, B7H6, is present in pSS salivary glands potentially triggering CCR9+ Th cells present in the glands, and in addition a SNP in this gene was associated with pSS potentially contributing to the pathogenesis (46). Identification of natural cytotoxicity receptor NCR3 on Th cells is unexpected, and indeed only a small percentage of CCR9+ Th cells expresses this molecule and levels are much higher on CD56+ NK cells (data not shown). Further investigation is needed to study whether this represents a functionally relevant expression by CCR9+ Th cells, potentially inducing IFN-γ production like in NK cells.

Elevated *NT5E* (CD73) gene expression was found in CCR9+ Th cells and on protein level CD73 (ecto-5’-nucleotidase) was expressed on 10-40% of CCR9+ Th cells. CD73 is an enzyme that dephosphorylates AMP into anti-inflammatory adenosine contributing to an anti-inflammatory milieu (63). CD73 is expressed by regulatory T cells but can be upregulated on all Th cells upon activation (64). As a homeostatic process, regulatory molecules are upregulated upon activation of T cells, and this may be the case for CD73 on the pro-inflammatory CCR9+ Th cells.

Another interesting molecule upregulated in a small subset of CCR9+ Th cells, is *ABCB1*, a multidrug resistance gene. It encodes for P-glycoprotein or MDR-1 (multidrug resistance 1) which causes efflux of intracellular drugs and is associated with unresponsiveness to treatment in various diseases including systemic lupus erythematosus (65). Some agents can induce expression of P-glycoprotein and some can inhibit its function, including cyclosporin-A. This suggests that increased expression of *ABCB1* on CCR9+ Th cells might contribute to resistance to inhibition by some drugs and might benefit from simultaneous inhibition of ABCB1 function (e.g. by cyclosporine-A) (66). Further research is needed to study the relevance of expression of *ABCB1* on CCR9+ Th cells and in pSS.

In some pSS patients, hypocomplementemia of in particular C3 and C4 is found, which is associated with lymphoma development (67). It is generally hypothesized that low C3 and C4 levels are a result of consumption mediated by immune complexes. This complement activation/consumption is likely associated with formation of complement fragments such as C3a and C4a, the latter being elevated in pSS salivary glands (68). Although not demonstrated on protein level or by functional experiments, *C3AR1* was found to be elevated in CCR9+ Th cells. Upregulated C3aR may contribute to CCR9+ Th cell activation since C3aR signaling has been shown to contribute to maintenance of effector functions *via* mTOR (69).

Although robust differences between Th subsets were detected and replicated, a drawback of our study is that the cohort in which RNA sequencing was performed was rather small. Especially since differences in mRNA expression between HC and pSS patients were small, the number of donors may not have been sufficient to detect true differences. The most robust differences between HC and pSS were present in all Th subsets; gene expression differences between HC and pSS that were specific for the CCR9+ Th cell subset were smaller. Indeed, the small CCR9+ Th cell subset-specific differences between HC and pSS patients were unfortunately not validated. False discovery rate correction may have partly increased the chance of replicating positive results from RNA sequencing. In this respect, DEGs from the modules showing the largest differences between HC and pSS patients i.e. cyan, darkgreen including IFN induced genes, which indeed have been shown to be upregulated in part of pSS patients (70), and purple, in which the differences between HC and pSS were present in all Th subsets, may have given a higher chance of being replicated in additional cohorts.

In addition, in this study circulating CCR9+ Th cells were studied, but whether these are recirculating from the gut or the salivary glands and to what extent these reflect tissue CCR9+ Th cells is unclear. As a future perspective, bulk or single cell sorting of CCR9+ Th cells from salivary glands of pSS patients to perform RNA sequencing and TCR sequencing may reveal the local activation profile and possible autoreactivity of this pro-inflammatory Th subset. Also, further analyses may elucidate the specific and shared molecular features as compared to ICOS+PD-1^hi^CXCR5- Tph cells (29, 30).

Also, variation in gene expression data may partly be due to patient heterogeneity. Indeed differences were observed between patients used for discovery (RNA sequencing) and replication (qPCR, flow cytometry and *in vitro*). A larger proportion of patients of the RNA sequencing cohort was treated with immunosuppressants and there were differences in clinical parameters (LFS, presence of auto-antibodies, ESSDAI scores, serum IgG levels). However, none of these clinical parameters showed statistically significant correlations with the expression of the target genes.

Finally, the already strongly differentiated nature of (re)circulating CCR9+ effector Th cells in any individual may also have hampered the detection of a difference between HC and pSS patients. Previously we have documented strongly increased percentages of IFN-γ, IL-17, IL-21 and IL-10-producing cells in circulating CCR9+ *vs* CXCR5+ Th cells (26), which was similar in HC and pSS patients. The increased numbers of CCR9+ Th cells in the circulation and the salivary glands suggest that given the effector functions of CCR9+ Th cells these may significantly contribute to immunopathology. In the same line, our *in vitro* data suggest that activation of CCR9+ Th cells upon antigen challenge and increased IL-7 significantly could drive local inflammation. Future research should elucidate the generalized differentiated nature of circulating CCR9+ effector Th cells in additional inflammatory diseases.

Altogether the present study reveals that CCR9+ Th cells show many differentially expressed genes as compared to CXCR5+ Th cells and CCR9-CXCR5- Th cells, identifying novel effector molecules that reveal additional properties of these pathogenic cells. This is exemplified by CCL5, which may be a key mediator in early migration of inflammatory cells. Targeting predicted key molecules based on the results from this study might reveal novel therapeutic approaches to halt the pathogenic processes induced by CCR9+ Th cells.

## Data Availability Statement

The RNA sequencing dataset generated for this study can be found in NCBI’s Gene Expression Omnibus (GEO) and is accessible through GEO Series accession number GSE173635. 

## Ethics Statement

The studies involving human participants were reviewed and approved by the Medical Research Ethics Committee (METC) of the University Medical Center Utrecht (approval METC no. 13/697). The patients/participants provided their written informed consent to participate in this study.

## Author Contributions

AH, SB, AL, AP, AK, TR, and JR were involved in conception and design of the study. AH, SB, CW, AL, AP, and AK were involved in data acquisition. AH, SB, AL, AK, AP, and JR were involved in data analysis and interpretation. AH drafted the manuscript. All authors contributed to the article and approved the submitted version.

## Funding

AH is supported by ReumaNederland (formerly Dutch Arthritis Association, grant number 17-2-403).

## Conflict of Interest

Currently TR is an employee of Abbvie where he holds stock. TR had no part in the design and interpretation of the study results after he started at Abbvie.

The remaining authors declare that the research was conducted in the absence of any commercial or financial relationships that could be construed as a potential conflict of interest.

## Publisher’s Note

All claims expressed in this article are solely those of the authors and do not necessarily represent those of their affiliated organizations, or those of the publisher, the editors and the reviewers. Any product that may be evaluated in this article, or claim that may be made by its manufacturer, is not guaranteed or endorsed by the publisher.
