# Supplementary material for: Transcriptome Analysis of CCR9+ T Helper Cells From Primary Sjögren’s Syndrome Patients Identifies CCL5 as a Novel Effector Molecule
Source: Front Immunol. 2021 Jul 27;12:702733. doi: 10.3389/fimmu.2021.702733 (PMC8354142; doi:10.3389/fimmu.2021.702733)
Supplement: Supplementary file 1 [file DataSheet_1.pdf]

Supplementary Material

Supplementary Table 1. Antibodies used for flow cytometry.

Marker	Fluorochrome	Company	Clone
**CD3**	AF700	Sony Biotechnology	UCHT1
**CD4**	PerCP	Biolegend	RPA-T4
	APC-ef780	eBioscience	RPA-T4
	BV785	Biolegend	RPA-T4
**CXCR5**	BV421	BD Biosciences	RF8B2
	PerCP-Cy5.5	Biolegend	J252D
**CCR9**	PE	R&D/Bio-Techne	248621
	APC	Biolegend	L053E8
**NKp30**	PE	BD Biosciences	P30-15
**CD8**	PerCP	BD Biosciences	SK1
	FITC	BD Biosciences	RPA-T8
**CD56**	FITC	Biolegend	HCD56
**CD73**	PE	BD Biosciences	AD2
**CD45RO**	BV711	Biolegend	UCHL1
	PE-Cy7	BD Biosciences	UCHL1
**CD27**	APC-ef780	eBioscience	O323
	BV510	BD Biosciences	L128
**CD103**	FITC	Dako	Ber-ACT8
**CD49a**	APC	Sony Biotechnology	TS2/7
**ABCB1**	FITC	eBioscience	UIC2
**CCR5**	PE	eBioscience	eBioT21/8
**CCL5**	PE	Biolegend	VL1
**IFN-gamma**	PerCP-Cy5.5	eBioscience	4S.B3
**TNF-alpha**	BV421	BD Biosciences	Mab11

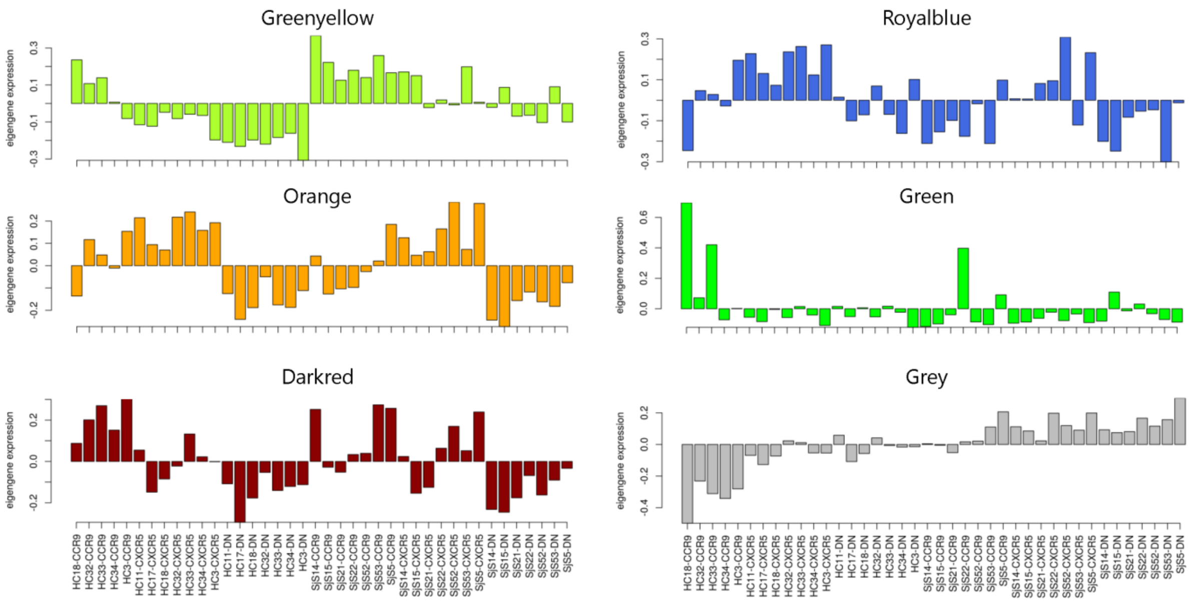

Supplementary figure 1. Excluded modules. Using WGCNA analysis 15 modules were constructed. Based on less robust patterns of distinct eigengene expression between Th cell subsets or between HC and pSS 6 modules were excluded from further analysis. The grey module contains genes which were not assigned to any module.

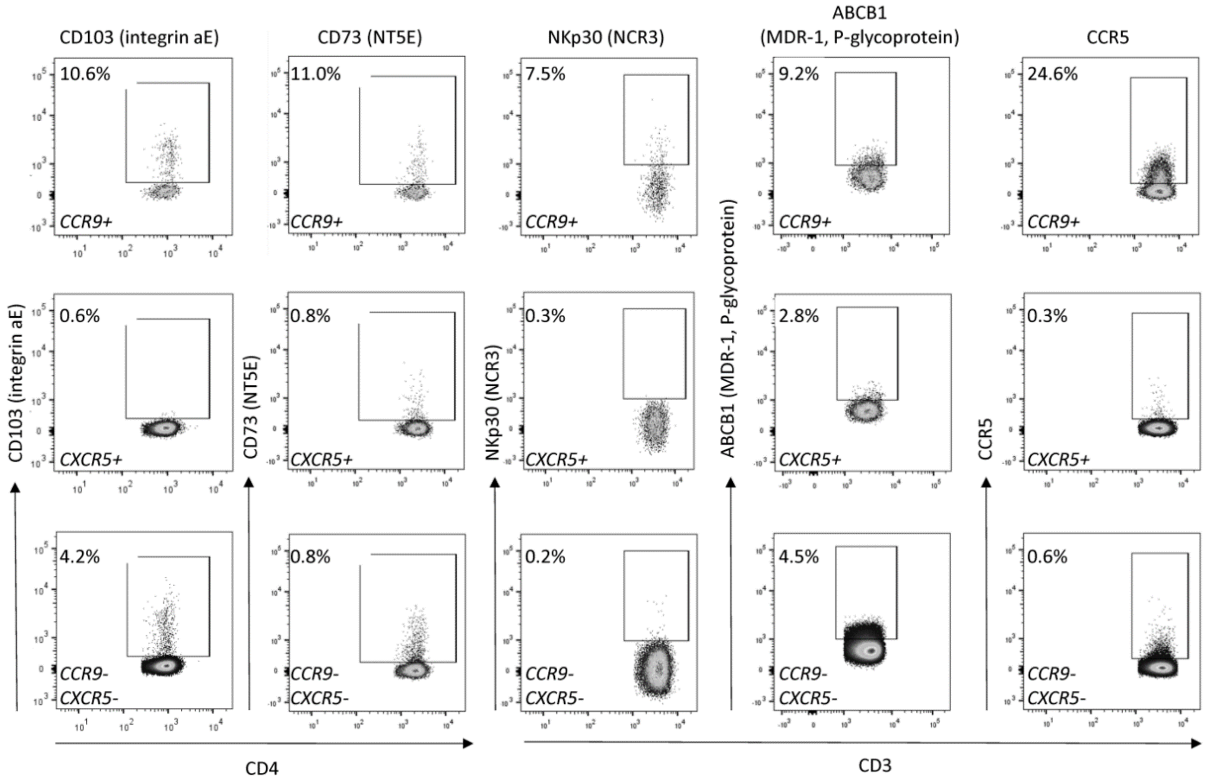

Supplementary figure 2. **Validation of target genes by flow cytometry**. Representative FACS stainings of CCR9+ Th cells (top row), CXCR5+ Th cells (middle row) and DN Th cells (last row) are shown for integrin αE (ITGAE), NKp30 (NCR3), CD73 (NT5E), ABCB1 (MDR-1, P-glycoprotein) and CCR5.

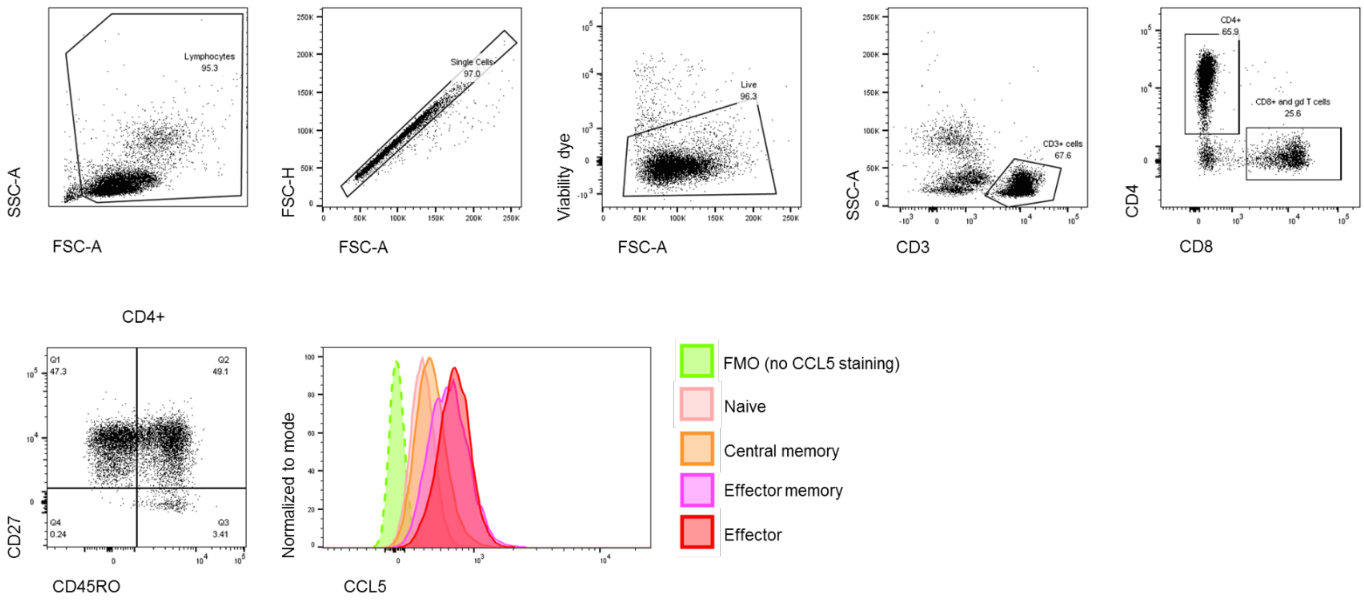

Supplementary figure 3. Gating strategy for intracellular flow cytometry staining of CCL5. Representative FACS staining of healthy control. Top row, panel left to right: forward versus side scatter (FSC vs SSC); selection of single cells, selection of live cells using fixable viability dye; selection of CD3+ cells, followed by selection of CD4+ cells. Bottom row: CD45RO/CD27 staining of CD4+ cells and CCL5 expression in given subsets.
